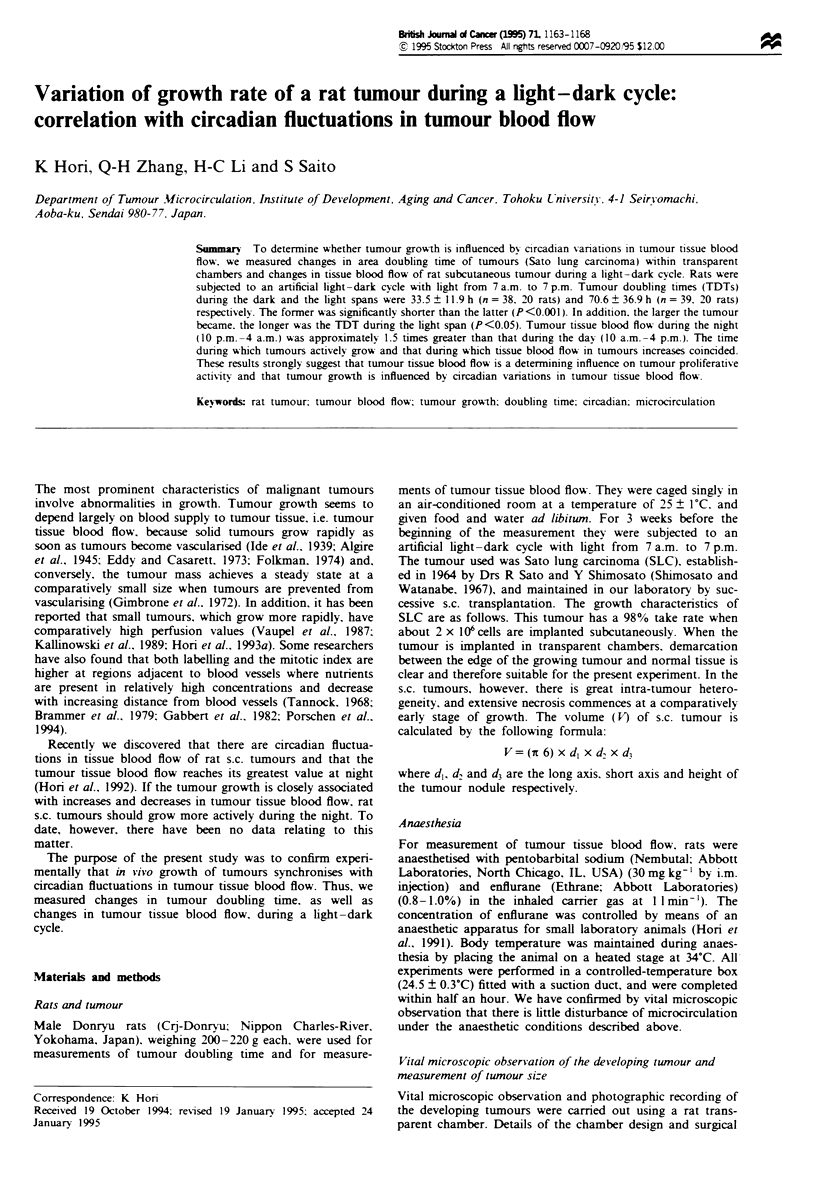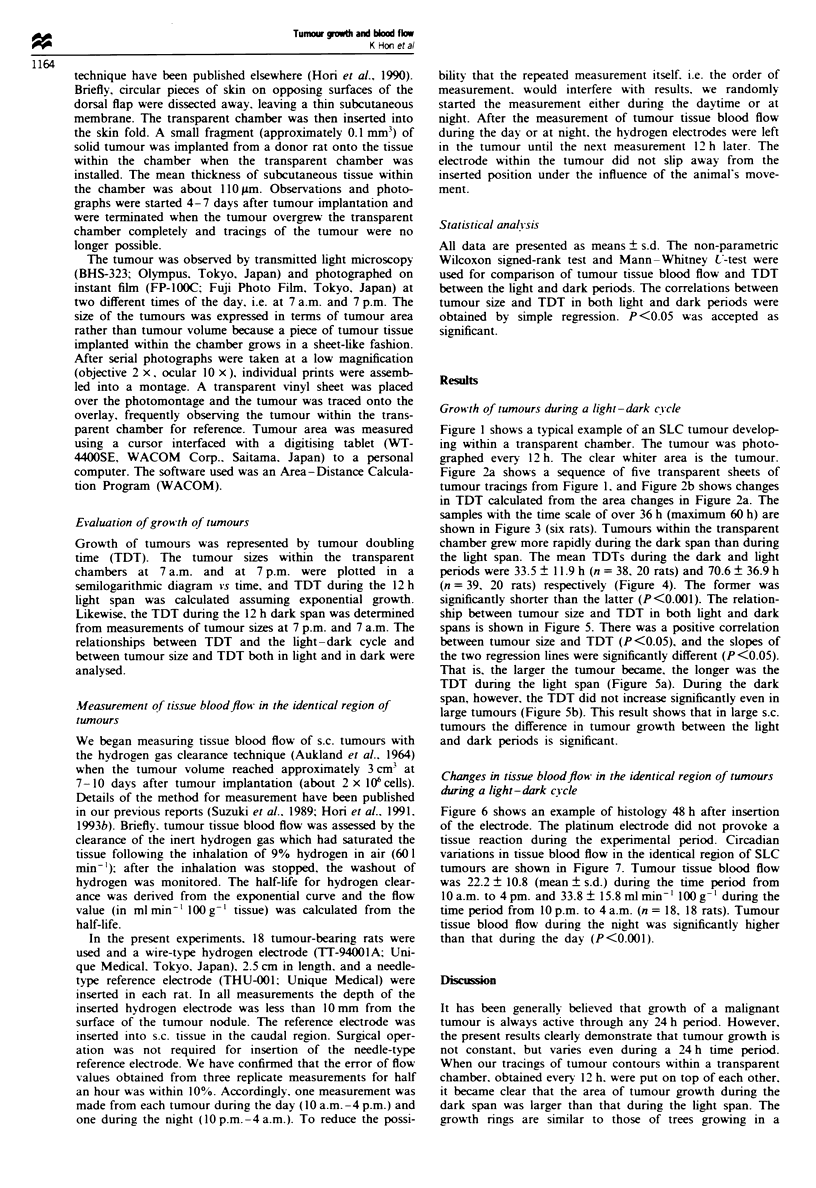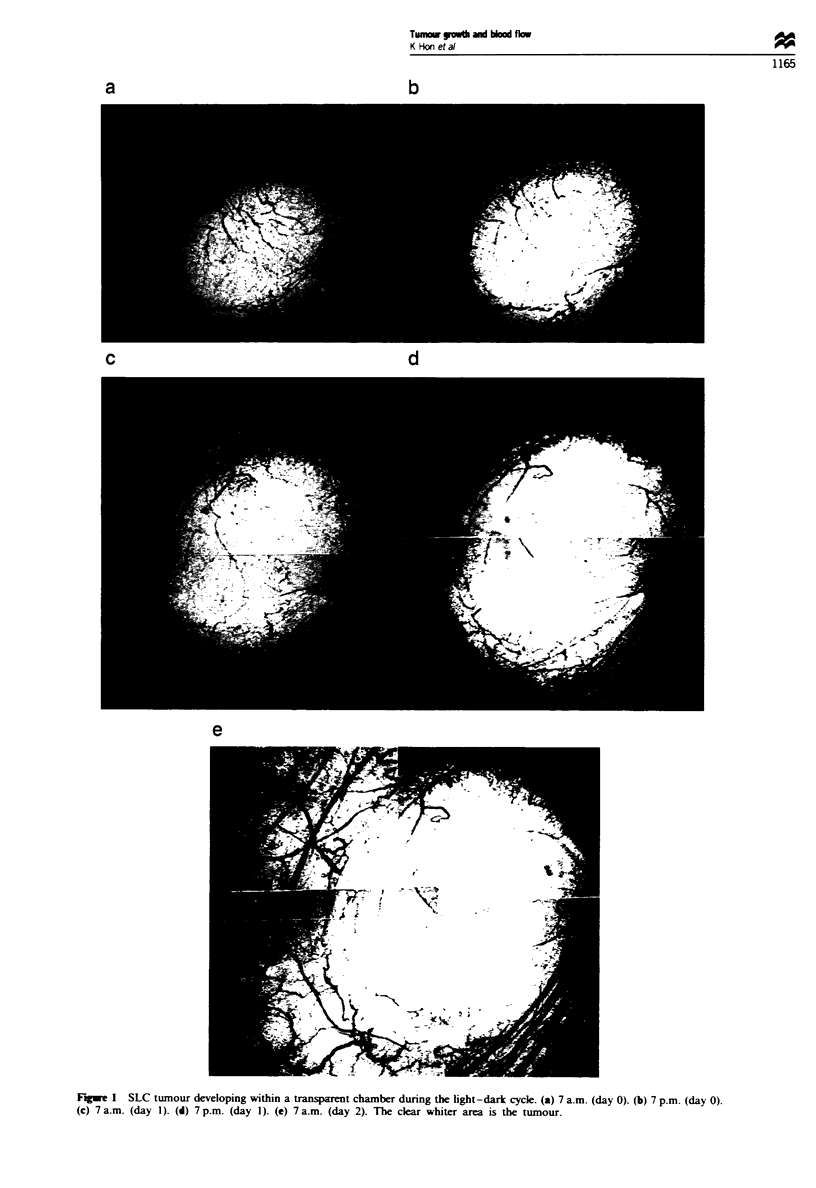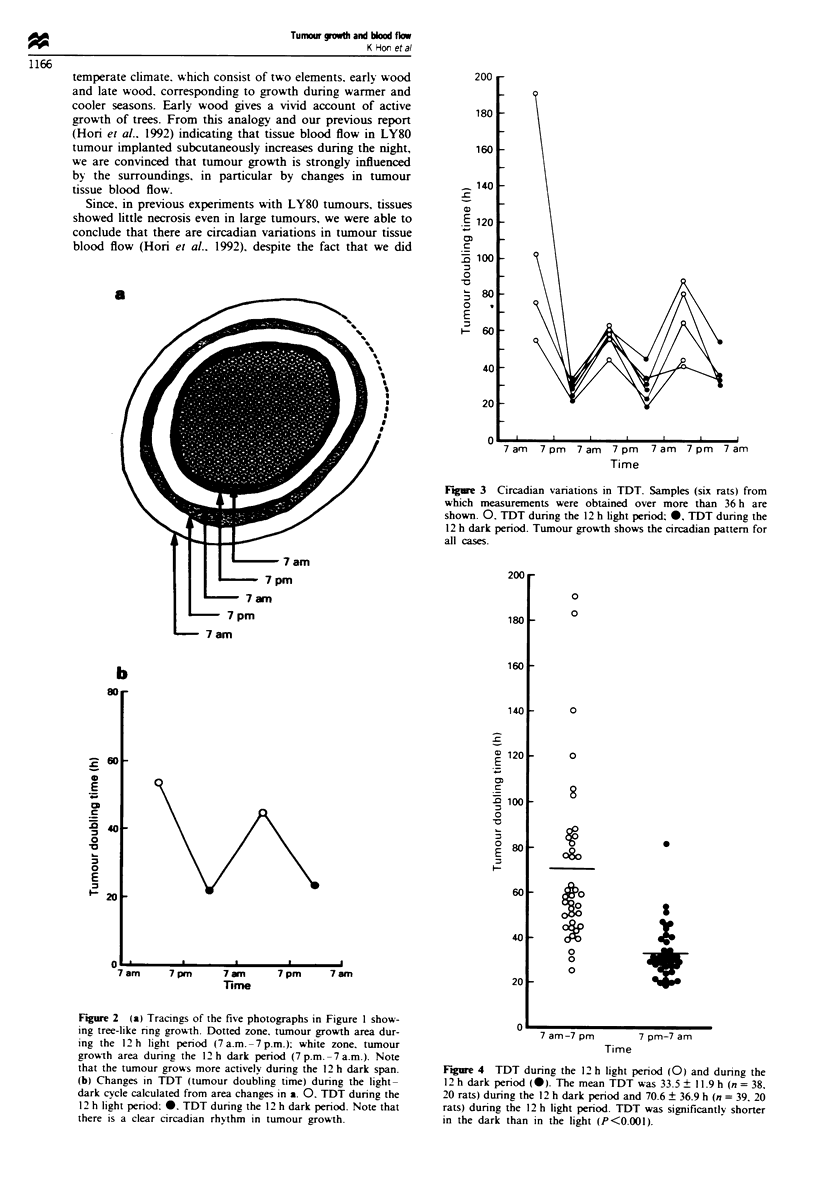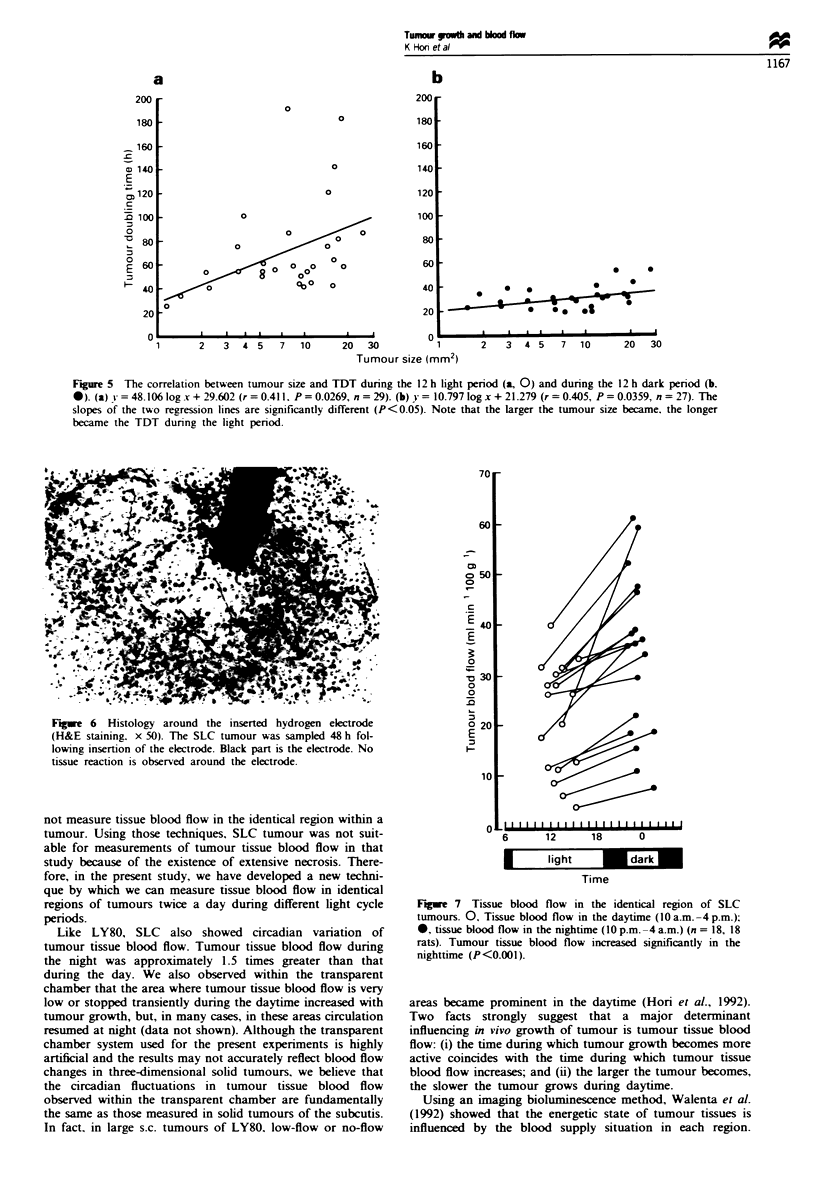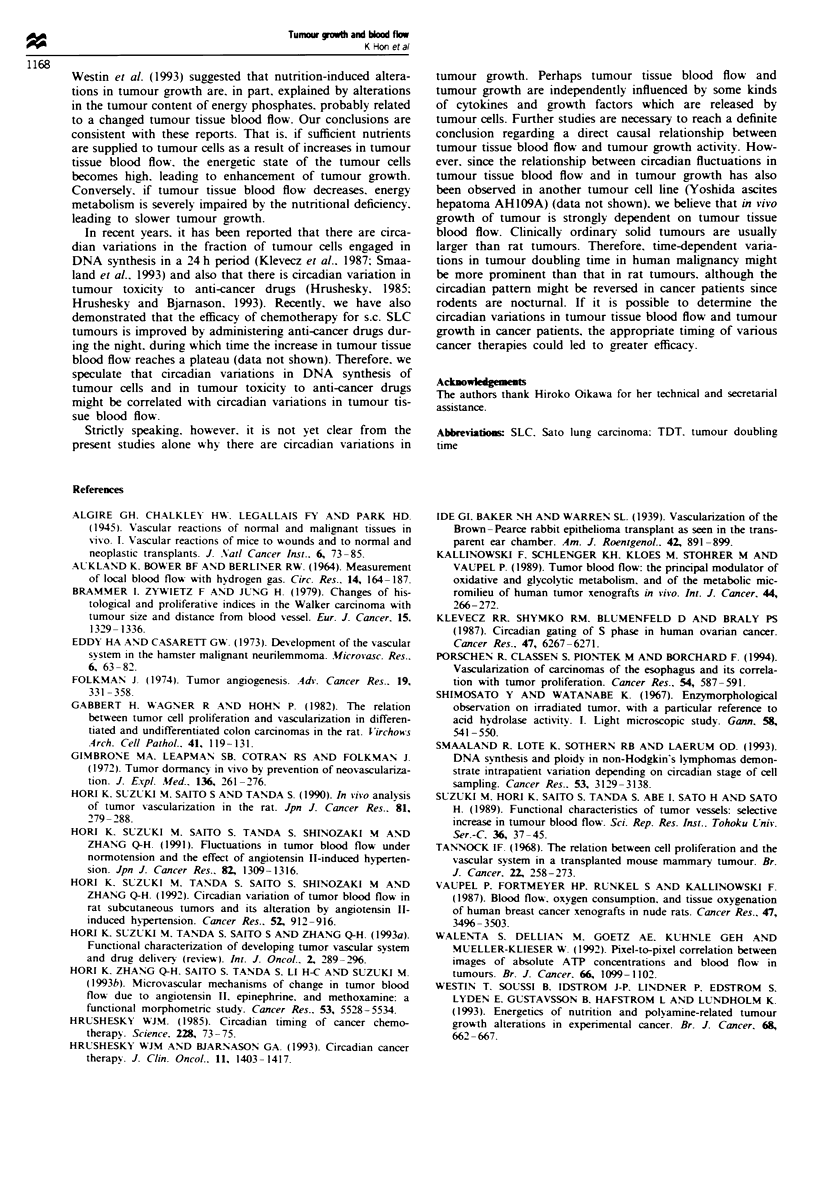# Variation of growth rate of a rat tumour during a light-dark cycle: correlation with circadian fluctuations in tumour blood flow.

**DOI:** 10.1038/bjc.1995.227

**Published:** 1995-06

**Authors:** K. Hori, Q. H. Zhang, H. C. Li, S. Saito

**Affiliations:** Department of Tumour Microcirculation, Tohoku University, Sendai, Japan.

## Abstract

**Images:**


© 1995 Stockton Press All rights reserved 0007-0920/95 $12.00

### Variation of growth rate of a rat tumour during a light-dark cycle: correlation with circadian fluctuations in tumour blood flow

K Hori, Q-H Zhang, H-C Li and S Saito

*Department of Tumour Microcirculation, Institute of Development, Aging and Cancer, Tohoku University, 4-1 Seiryomachi, Aoba-ku, Sendai 980-77, Japan.*

**Summary** To determine whether tumour growth is influenced by circadian variations in tumour tissue blood flow, we measured changes in area doubling time of tumours (Sato lung carcinoma) within transparent chambers and changes in tissue blood flow of rat subcutaneous tumour during a light-dark cycle. Rats were subjected to an artificial light-dark cycle with light from 7 a.m. to 7 p.m. Tumour doubling times (TDTs) during the dark and the light spans were 33.5 ± 11.9 h ($n = 38$, 20 rats) and 70.6 ± 36.9 h ($n = 39$, 20 rats) respectively. The former was significantly shorter than the latter ($P < 0.001$). In addition, the larger the tumour became, the longer was the TDT during the light span ($P < 0.05$). Tumour tissue blood flow during the night (10 p.m.-4 a.m.) was approximately 1.5 times greater than that during the day (10 a.m.-4 p.m.). The time during which tumours actively grow and that during which tissue blood flow in tumours increases coincided. These results strongly suggest that tumour tissue blood flow is a determining influence on tumour proliferative activity and that tumour growth is influenced by circadian variations in tumour tissue blood flow.

**Keywords:** rat tumour; tumour blood flow; tumour growth; doubling time; circadian; microcirculation

The most prominent characteristics of malignant tumours involve abnormalities in growth. Tumour growth seems to depend largely on blood supply to tumour tissue, i.e. tumour tissue blood flow, because solid tumours grow rapidly as soon as tumours become vascularised (Ide *et al.*, 1939; Algire *et al.*, 1945; Eddy and Casarett, 1973; Folkman, 1974) and, conversely, the tumour mass achieves a steady state at a comparatively small size when tumours are prevented from vascularising (Gimbrone *et al.*, 1972). In addition, it has been reported that small tumours, which grow more rapidly, have comparatively high perfusion values (Vaupel *et al.*, 1987; Kallinowski *et al.*, 1989; Hori *et al.*, 1993*a*). Some researchers have also found that both labelling and the mitotic index are higher at regions adjacent to blood vessels where nutrients are present in relatively high concentrations and decrease with increasing distance from blood vessels (Tannock, 1968; Brammer *et al.*, 1979; Gabbert *et al.*, 1982; Porschen *et al.*, 1994).

Recently we discovered that there are circadian fluctuations in tissue blood flow of rat s.c. tumours and that the tumour tissue blood flow reaches its greatest value at night (Hori *et al.*, 1992). If the tumour growth is closely associated with increases and decreases in tumour tissue blood flow, rat s.c. tumours should grow more actively during the night. To date, however, there have been no data relating to this matter.

The purpose of the present study was to confirm experimentally that *in vivo* growth of tumours synchronises with circadian fluctuations in tumour tissue blood flow. Thus, we measured changes in tumour doubling time, as well as changes in tumour tissue blood flow, during a light-dark cycle.

### Materials and methods

### Rats and tumour

Male Donryu rats (Crj-Donryu; Nippon Charles-River, Yokohama, Japan), weighing 200-220 g each, were used for measurements of tumour doubling time and for measurements of tumour tissue blood flow. They were caged singly in an air-conditioned room at a temperature of 25 ± 1°C, and given food and water *ad libitum*. For 3 weeks before the beginning of the measurement they were subjected to an artificial light-dark cycle with light from 7 a.m. to 7 p.m. The tumour used was Sato lung carcinoma (SLC), established in 1964 by Drs R Sato and Y Shimosato (Shimosato and Watanabe, 1967), and maintained in our laboratory by successive s.c. transplantation. The growth characteristics of SLC are as follows. This tumour has a 98% take rate when about $2 \times 10^6$ cells are implanted subcutaneously. When the tumour is implanted in transparent chambers, demarcation between the edge of the growing tumour and normal tissue is clear and therefore suitable for the present experiment. In the s.c. tumours, however, there is great intra-tumour heterogeneity, and extensive necrosis commences at a comparatively early stage of growth. The volume ($V$) of s.c. tumour is calculated by the following formula:

$$V = (\pi/6) \times d_1 \times d_2 \times d_3$$

where $d_1$, $d_2$ and $d_3$ are the long axis, short axis and height of the tumour nodule respectively.

### Anaesthesia

For measurement of tumour tissue blood flow, rats were anaesthetised with pentobarbital sodium (Nembutal; Abbott Laboratories, North Chicago, IL, USA) (30 mg kg$^{-1}$ by i.m. injection) and enflurane (Ethrane; Abbott Laboratories) (0.8-1.0%) in the inhaled carrier gas at 1 l min$^{-1}$). The concentration of enflurane was controlled by means of an anaesthetic apparatus for small laboratory animals (Hori *et al.*, 1991). Body temperature was maintained during anaesthesia by placing the animal on a heated stage at 34°C. All experiments were performed in a controlled-temperature box (24.5 ± 0.3°C) fitted with a suction duct, and were completed within half an hour. We have confirmed by vital microscopic observation that there is little disturbance of microcirculation under the anaesthetic conditions described above.

### Vital microscopic observation of the developing tumour and measurement of tumour size

Vital microscopic observation and photographic recording of the developing tumours were carried out using a rat transparent chamber. Details of the chamber design and surgical

Correspondence: K Hori
Received 19 October 1994; revised 19 January 1995; accepted 24 January 1995

technique have been published elsewhere (Hori *et al.*, 1990). Briefly, circular pieces of skin on opposing surfaces of the dorsal flap were dissected away, leaving a thin subcutaneous membrane. The transparent chamber was then inserted into the skin fold. A small fragment (approximately 0.1 $mm^3$) of solid tumour was implanted from a donor rat onto the tissue within the chamber when the transparent chamber was installed. The mean thickness of subcutaneous tissue within the chamber was about 110 $\mu$m. Observations and photographs were started 4–7 days after tumour implantation and were terminated when the tumour overgrew the transparent chamber completely and tracings of the tumour were no longer possible.

The tumour was observed by transmitted light microscopy (BHS-323; Olympus, Tokyo, Japan) and photographed on instant film (FP-100C; Fuji Photo Film, Tokyo, Japan) at two different times of the day, i.e. at 7 a.m. and 7 p.m. The size of the tumours was expressed in terms of tumour area rather than tumour volume because a piece of tumour tissue implanted within the chamber grows in a sheet-like fashion. After serial photographs were taken at a low magnification (objective 2 ×, ocular 10 ×), individual prints were assembled into a montage. A transparent vinyl sheet was placed over the photomontage and the tumour was traced onto the overlay, frequently observing the tumour within the transparent chamber for reference. Tumour area was measured using a cursor interfaced with a digitising tablet (WT-4400SE, WACOM Corp., Saitama, Japan) to a personal computer. The software used was an Area–Distance Calculation Program (WACOM).

### *Evaluation of growth of tumours*

Growth of tumours was represented by tumour doubling time (TDT). The tumour sizes within the transparent chambers at 7 a.m. and at 7 p.m. were plotted in a semilogarithmic diagram *vs* time, and TDT during the 12 h light span was calculated assuming exponential growth. Likewise, the TDT during the 12 h dark span was determined from measurements of tumour sizes at 7 p.m. and 7 a.m. The relationships between TDT and the light–dark cycle and between tumour size and TDT both in light and in dark were analysed.

### *Measurement of tissue blood flow in the identical region of tumours*

We began measuring tissue blood flow of s.c. tumours with the hydrogen gas clearance technique (Aukland *et al.*, 1964) when the tumour volume reached approximately 3 $cm^3$ at 7–10 days after tumour implantation (about $2 \times 10^6$ cells). Details of the method for measurement have been published in our previous reports (Suzuki *et al.*, 1989; Hori *et al.*, 1991, 1993*b*). Briefly, tumour tissue blood flow was assessed by the clearance of the inert hydrogen gas which had saturated the tissue following the inhalation of 9% hydrogen in air (60 l $min^{-1}$); after the inhalation was stopped, the washout of hydrogen was monitored. The half-life for hydrogen clearance was derived from the exponential curve and the flow value (in ml $min^{-1}$ 100 $g^{-1}$ tissue) was calculated from the half-life.

In the present experiments, 18 tumour-bearing rats were used and a wire-type hydrogen electrode (TT-94001A; Unique Medical, Tokyo, Japan), 2.5 cm in length, and a needle-type reference electrode (THU-001; Unique Medical) were inserted in each rat. In all measurements the depth of the inserted hydrogen electrode was less than 10 mm from the surface of the tumour nodule. The reference electrode was inserted into s.c. tissue in the caudal region. Surgical operation was not required for insertion of the needle-type reference electrode. We have confirmed that the error of flow values obtained from three replicate measurements for half an hour was within 10%. Accordingly, one measurement was made from each tumour during the day (10 a.m.–4 p.m.) and one during the night (10 p.m.–4 a.m.). To reduce the possibility that the repeated measurement itself, i.e. the order of measurement, would interfere with results, we randomly started the measurement either during the daytime or at night. After the measurement of tumour tissue blood flow during the day or at night, the hydrogen electrodes were left in the tumour until the next measurement 12 h later. The electrode within the tumour did not slip away from the inserted position under the influence of the animal's movement.

### *Statistical analysis*

All data are presented as means ± s.d. The non-parametric Wilcoxon signed-rank test and Mann–Whitney *U*-test were used for comparison of tumour tissue blood flow and TDT between the light and dark periods. The correlations between tumour size and TDT in both light and dark periods were obtained by simple regression. $P < 0.05$ was accepted as significant.

### Results

### *Growth of tumours during a light–dark cycle*

Figure 1 shows a typical example of an SLC tumour developing within a transparent chamber. The tumour was photographed every 12 h. The clear whiter area is the tumour. Figure 2a shows a sequence of five transparent sheets of tumour tracings from Figure 1, and Figure 2b shows changes in TDT calculated from the area changes in Figure 2a. The samples with the time scale of over 36 h (maximum 60 h) are shown in Figure 3 (six rats). Tumours within the transparent chamber grew more rapidly during the dark span than during the light span. The mean TDTs during the dark and light periods were 33.5 ± 11.9 h ($n = 38$, 20 rats) and 70.6 ± 36.9 h ($n = 39$, 20 rats) respectively (Figure 4). The former was significantly shorter than the latter ($P < 0.001$). The relationship between tumour size and TDT in both light and dark spans is shown in Figure 5. There was a positive correlation between tumour size and TDT ($P < 0.05$), and the slopes of the two regression lines were significantly different ($P < 0.05$). That is, the larger the tumour became, the longer was the TDT during the light span (Figure 5a). During the dark span, however, the TDT did not increase significantly even in large tumours (Figure 5b). This result shows that in large s.c. tumours the difference in tumour growth between the light and dark periods is significant.

### *Changes in tissue blood flow in the identical region of tumours during a light–dark cycle*

Figure 6 shows an example of histology 48 h after insertion of the electrode. The platinum electrode did not provoke a tissue reaction during the experimental period. Circadian variations in tissue blood flow in the identical region of SLC tumours are shown in Figure 7. Tumour tissue blood flow was 22.2 ± 10.8 (mean ± s.d.) during the time period from 10 a.m. to 4 pm. and 33.8 ± 15.8 ml $min^{-1}$ 100 $g^{-1}$ during the time period from 10 p.m. to 4 a.m. ($n = 18$, 18 rats). Tumour tissue blood flow during the night was significantly higher than that during the day ($P < 0.001$).

### Discussion

It has been generally believed that growth of a malignant tumour is always active through any 24 h period. However, the present results clearly demonstrate that tumour growth is not constant, but varies even during a 24 h time period. When our tracings of tumour contours within a transparent chamber, obtained every 12 h, were put on top of each other, it became clear that the area of tumour growth during the dark span was larger than that during the light span. The growth rings are similar to those of trees growing in a

**Figure 1** SLC tumour developing within a transparent chamber during the light–dark cycle. (**a**) 7 a.m. (day 0). (**b**) 7 p.m. (day 0). (**c**) 7 a.m. (day 1). (**d**) 7 p.m. (day 1). (**e**) 7 a.m. (day 2). The clear whiter area is the tumour.

temperate climate, which consist of two elements, early wood and late wood, corresponding to growth during warmer and cooler seasons. Early wood gives a vivid account of active growth of trees. From this analogy and our previous report (Hori *et al.*, 1992) indicating that tissue blood flow in LY80 tumour implanted subcutaneously increases during the night, we are convinced that tumour growth is strongly influenced by the surroundings, in particular by changes in tumour tissue blood flow.

Since, in previous experiments with LY80 tumours, tissues showed little necrosis even in large tumours, we were able to conclude that there are circadian variations in tumour tissue blood flow (Hori *et al.*, 1992), despite the fact that we did

**Figure 2** (**a**) Tracings of the five photographs in Figure 1 showing tree-like ring growth. Dotted zone, tumour growth area during the 12 h light period (7 a.m. - 7 p.m.); white zone, tumour growth area during the 12 h dark period (7 p.m. - 7 a.m.). Note that the tumour grows more actively during the 12 h dark span. (**b**) Changes in TDT (tumour doubling time) during the light-dark cycle calculated from area changes in **a**. ○, TDT during the 12 h light period; ●, TDT during the 12 h dark period. Note that there is a clear circadian rhythm in tumour growth.

**Figure 3** Circadian variations in TDT. Samples (six rats) from which measurements were obtained over more than 36 h are shown. ○, TDT during the 12 h light period; ●, TDT during the 12 h dark period. Tumour growth shows the circadian pattern for all cases.

**Figure 4** TDT during the 12 h light period (○) and during the 12 h dark period (●). The mean TDT was 33.5 ± 11.9 h ($n$ = 38, 20 rats) during the 12 h dark period and 70.6 ± 36.9 h ($n$ = 39, 20 rats) during the 12 h light period. TDT was significantly shorter in the dark than in the light ($P$<0.001).

**Figure 5** The correlation between tumour size and TDT during the 12 h light period (**a**, O) and during the 12 h dark period (**b**, ●). (**a**) $y = 48.106 \log x + 29.602$ ($r = 0.411$, $P = 0.0269$, $n = 29$). (**b**) $y = 10.797 \log x + 21.279$ ($r = 0.405$, $P = 0.0359$, $n = 27$). The slopes of the two regression lines are significantly different ($P < 0.05$). Note that the larger the tumour size became, the longer became the TDT during the light period.

**Figure 6** Histology around the inserted hydrogen electrode (H&E staining, × 50). The SLC tumour was sampled 48 h following insertion of the electrode. Black part is the electrode. No tissue reaction is observed around the electrode.

**Figure 7** Tissue blood flow in the identical region of SLC tumours. O, Tissue blood flow in the daytime (10 a.m.–4 p.m.); ●, tissue blood flow in the nightime (10 p.m.–4 a.m.) ($n$ = 18, 18 rats). Tumour tissue blood flow increased significantly in the nighttime ($P < 0.001$).

not measure tissue blood flow in the identical region within a tumour. Using those techniques, SLC tumour was not suitable for measurements of tumour tissue blood flow in that study because of the existence of extensive necrosis. Therefore, in the present study, we have developed a new technique by which we can measure tissue blood flow in identical regions of tumours twice a day during different light cycle periods.

Like LY80, SLC also showed circadian variation of tumour tissue blood flow. Tumour tissue blood flow during the night was approximately 1.5 times greater than that during the day. We also observed within the transparent chamber that the area where tumour tissue blood flow is very low or stopped transiently during the daytime increased with tumour growth, but, in many cases, in these areas circulation resumed at night (data not shown). Although the transparent chamber system used for the present experiments is highly artificial and the results may not accurately reflect blood flow changes in three-dimensional solid tumours, we believe that the circadian fluctuations in tumour tissue blood flow observed within the transparent chamber are fundamentally the same as those measured in solid tumours of the subcutis. In fact, in large s.c. tumours of LY80, low-flow or no-flow areas became prominent in the daytime (Hori *et al.*, 1992). Two facts strongly suggest that a major determinant influencing *in vivo* growth of tumour is tumour tissue blood flow: (i) the time during which tumour growth becomes more active coincides with the time during which tumour tissue blood flow increases; and (ii) the larger the tumour becomes, the slower the tumour grows during daytime.

Using an imaging bioluminescence method, Walenta *et al.* (1992) showed that the energetic state of tumour tissues is influenced by the blood supply situation in each region.

Westin et al. (1993) suggested that nutrition-induced alterations in tumour growth are, in part, explained by alterations in the tumour content of energy phosphates, probably related to a changed tumour tissue blood flow. Our conclusions are consistent with these reports. That is, if sufficient nutrients are supplied to tumour cells as a result of increases in tumour tissue blood flow, the energetic state of the tumour cells becomes high, leading to enhancement of tumour growth. Conversely, if tumour tissue blood flow decreases, energy metabolism is severely impaired by the nutritional deficiency, leading to slower tumour growth.

In recent years, it has been reported that there are circadian variations in the fraction of tumour cells engaged in DNA synthesis in a 24 h period (Klevecz et al., 1987; Smaaland et al., 1993) and also that there is circadian variation in tumour toxicity to anti-cancer drugs (Hrushesky, 1985; Hrushesky and Bjarnason, 1993). Recently, we have also demonstrated that the efficacy of chemotherapy for s.c. SLC tumours is improved by administering anti-cancer drugs during the night, during which time the increase in tumour tissue blood flow reaches a plateau (data not shown). Therefore, we speculate that circadian variations in DNA synthesis of tumour cells and in tumour toxicity to anti-cancer drugs might be correlated with circadian variations in tumour tissue blood flow.

Strictly speaking, however, it is not yet clear from the present studies alone why there are circadian variations in tumour growth. Perhaps tumour tissue blood flow and tumour growth are independently influenced by some kinds of cytokines and growth factors which are released by tumour cells. Further studies are necessary to reach a definite conclusion regarding a direct causal relationship between tumour tissue blood flow and tumour growth activity. However, since the relationship between circadian fluctuations in tumour tissue blood flow and in tumour growth has also been observed in another tumour cell line (Yoshida ascites hepatoma AH109A) (data not shown), we believe that *in vivo* growth of tumour is strongly dependent on tumour tissue blood flow. Clinically ordinary solid tumours are usually larger than rat tumours. Therefore, time-dependent variations in tumour doubling time in human malignancy might be more prominent than that in rat tumours, although the circadian pattern might be reversed in cancer patients since rodents are nocturnal. If it is possible to determine the circadian variations in tumour tissue blood flow and tumour growth in cancer patients, the appropriate timing of various cancer therapies could led to greater efficacy.

**Acknowledgements**
The authors thank Hiroko Oikawa for her technical and secretarial assistance.

**Abbreviations:** SLC, Sato lung carcinoma; TDT, tumour doubling time

**References**

ALGIRE GH, CHALKLEY HW, LEGALLAIS FY AND PARK HD. (1945). Vascular reactions of normal and malignant tissues in vivo. I. Vascular reactions of mice to wounds and to normal and neoplastic transplants. *J. Natl Cancer Inst.*, **6**, 73–85.

AUKLAND K, BOWER BF AND BERLINER RW. (1964). Measurement of local blood flow with hydrogen gas. *Circ. Res.*, **14**, 164–187.

BRAMMER I, ZYWIETZ F AND JUNG H. (1979). Changes of histological and proliferative indices in the Walker carcinoma with tumour size and distance from blood vessel. *Eur. J. Cancer*, **15**, 1329–1336.

EDDY HA AND CASARETT GW. (1973). Development of the vascular system in the hamster malignant neurilemmoma. *Microvasc. Res.*, **6**, 63–82.

FOLKMAN J. (1974). Tumor angiogenesis. *Adv. Cancer Res.*, **19**, 331–358.

GABBERT H, WAGNER R AND HOHN P. (1982). The relation between tumor cell proliferation and vascularization in differentiated and undifferentiated colon carcinomas in the rat. *Virchows Arch. Cell Pathol.*, **41**, 119–131.

GIMBRONE MA, LEAPMAN SB, COTRAN RS AND FOLKMAN J. (1972). Tumor dormancy in vivo by prevention of neovascularization. *J. Expl. Med.*, **136**, 261–276.

HORI K, SUZUKI M, SAITO S AND TANDA S. (1990). *In vivo* analysis of tumor vascularization in the rat. *Jpn J. Cancer Res.*, **81**, 279–288.

HORI K, SUZUKI M, SAITO S, TANDA S, SHINOZAKI M AND ZHANG Q-H. (1991). Fluctuations in tumor blood flow under normotension and the effect of angiotensin II-induced hypertension. *Jpn J. Cancer Res.*, **82**, 1309–1316.

HORI K, SUZUKI M, TANDA S, SAITO S, SHINOZAKI M AND ZHANG Q-H. (1992). Circadian variation of tumor blood flow in rat subcutaneous tumors and its alteration by angiotensin II-induced hypertension. *Cancer Res.*, **52**, 912–916.

HORI K, SUZUKI M, TANDA S, SAITO S AND ZHANG Q-H. (1993*a*). Functional characterization of developing tumor vascular system and drug delivery (review). *Int. J. Oncol.*, **2**, 289–296.

HORI K, ZHANG Q-H, SAITO S, TANDA S, LI H-C AND SUZUKI M. (1993*b*). Microvascular mechanisms of change in tumor blood flow due to angiotensin II, epinephrine, and methoxamine: a functional morphometric study. *Cancer Res.*, **53**, 5528–5534.

HRUSHESKY WJM. (1985). Circadian timing of cancer chemotherapy. *Science*, **228**, 73–75.

HRUSHESKY WJM AND BJARNASON GA. (1993). Circadian cancer therapy. *J. Clin. Oncol.*, **11**, 1403–1417.

IDE GI, BAKER NH AND WARREN SL. (1939). Vascularization of the Brown–Pearce rabbit epithelioma transplant as seen in the transparent ear chamber. *Am. J. Roentgenol.*, **42**, 891–899.

KALLINOWSKI F, SCHLENGER KH, KLOES M, STOHRER M AND VAUPEL P. (1989). Tumor blood flow: the principal modulator of oxidative and glycolytic metabolism, and of the metabolic micromilieu of human tumor xenografts *in vivo*. *Int. J. Cancer*, **44**, 266–272.

KLEVECZ RR, SHYMKO RM, BLUMENFELD D AND BRALY PS (1987). Circadian gating of S phase in human ovarian cancer. *Cancer Res.*, **47**, 6267–6271.

PORSCHEN R, CLASSEN S, PIONTEK M AND BORCHARD F. (1994). Vascularization of carcinomas of the esophagus and its correlation with tumor proliferation. *Cancer Res.*, **54**, 587–591.

SHIMOSATO Y AND WATANABE K. (1967). Enzymorphological observation on irradiated tumor, with a particular reference to acid hydrolase activity. I. Light microscopic study. *Gann*, **58**, 541–550.

SMAALAND R, LOTE K, SOTHERN RB AND LAERUM OD. (1993). DNA synthesis and ploidy in non-Hodgkin's lymphomas demonstrate intrapatient variation depending on circadian stage of cell sampling. *Cancer Res.*, **53**, 3129–3138.

SUZUKI M, HORI K, SAITO S, TANDA S, ABE I, SATO H AND SATO H. (1989). Functional characteristics of tumor vessels: selective increase in tumour blood flow. *Sci. Rep. Res. Inst., Tohoku Univ. Ser.-C*, **36**, 37–45.

TANNOCK IF. (1968). The relation between cell proliferation and the vascular system in a transplanted mouse mammary tumour. *Br. J. Cancer*, **22**, 258–273.

VAUPEL P, FORTMEYER HP, RUNKEL S AND KALLINOWSKI F. (1987). Blood flow, oxygen consumption, and tissue oxygenation of human breast cancer xenografts in nude rats. *Cancer Res.*, **47**, 3496–3503.

WALENTA S, DELLIAN M, GOETZ AE, KUHNLE GEH AND MUELLER-KLIESER W. (1992). Pixel-to-pixel correlation between images of absolute ATP concentrations and blood flow in tumours. *Br. J. Cancer*, **66**, 1099–1102.

WESTIN T, SOUSSI B, IDSTROM J-P, LINDNER P, EDSTROM S, LYDEN E, GUSTAVSSON B, HAFSTROM L AND LUNDHOLM K. (1993). Energetics of nutrition and polyamine-related tumour growth alterations in experimental cancer. *Br. J. Cancer*, **68**, 662–667.